# Gestational exposure to the artificial sweetener erythritol reprograms ovarian function through AMH suppression and oxidative stress-mediated disruption of autophagy and PI3K signaling

**DOI:** 10.3389/fendo.2026.1829421

**Published:** 2026-05-14

**Authors:** Amina Fallata, Saber Nahdi, Hassan S. Alamri, Tlili Barhoumi, Mohamed Alanazi, Md Ataur Rahman, Saleh Alwasel, Abdel Halim Harrath

**Affiliations:** 1 Department of Zoology, College of Science, King Saud University, Riyadh, Saudi Arabia; 2 Department of Clinical Laboratory Sciences, College of Applied Medical Sciences, King Abdullah International Medical Research Center, King Saud bin Abdulaziz University for Health Sciences, Ministry of the National Guard-Health Affairs, Riyadh, Saudi Arabia; 3 Core Lab Facility, King Abdullah International Medical Research Center, King Saud bin Abdulaziz University for Health Sciences, Ministry of the National Guard-Health Affairs, Riyadh, Saudi Arabia; 4 Department of Biochemistry, College of Science, King Saud University, Riyadh, Saudi Arabia; 5 Department of Neurology, University of Michigan, Ann Arbor, MI, United States

**Keywords:** aging, autophagy, erythritol, ovary, oxidative stress, PI3K signaling, sweeteners

## Abstract

**Objective:**

Erythritol is a widely used non-nutritive sweetener generally regarded as safe. However, recent emerging evidence has begun to reveal potential adverse effects, including possible associations with cancer and cardiovascular disease. Nevertheless, its potential impact on female reproductive health remains largely unexplored.

**Methods:**

The present study investigated the transgenerational effects of gestational erythritol exposure on ovarian morphology and molecular signaling in Wistar rats. Pregnant females were administered 0.4 or 4 g/kg/day of erythritol from gestational day 6 until parturition. Ovarian histopathological architecture along with oxidative stress and molecular signaling markers were evaluated in F1 and F2 female offspring at the prepubertal stage.

**Results:**

We found that *in utero* erythritol exposure markedly disrupted folliculogenesis, increasing primordial follicle counts while significantly reducing Graafian follicles. Histological examination revealed notable anomalies, including degenerating and multi-oocyte follicles and granulosa cell pyknosis. Oxidative stress was significantly elevated across generations, as evidenced by increased malondialdehyde (MDA) levels and reduced superoxide dismutase (SOD) and glutathione (GSH) activity. Anti-Müllerian hormone (AMH) was significantly diminished, indicative of impaired granulosa cell functionality and reduced follicular competence. Autophagy markers, LC3 and Atg5, were markedly suppressed in F2 offspring, strongly implicating a transgenerational susceptibility mechanism potentially attributable to epigenetic reprogramming, given that global DNA methylation levels were significantly reduced in F2 offspring. Furthermore, elevated erythritol levels in F2 offspring were associated with reduced PI3K–p85 expression, indicating compromised follicular survival signaling.

**Discussion:**

Collectively, these findings demonstrate that gestational erythritol exposure promotes oxidative stress, disrupts autophagy, impairs steroidogenesis, and inhibits PI3K signaling, culminating in transgenerational ovarian dysfunction.

## 1 Introduction

The female infertility burden exhibited a concerning upward trend over recent decades (1). In 2021, approximately 110 million women were living with infertility compared with 55 million men (2). Infertility is a highly heterogeneous pathological condition with a multifactorial etiology, including genetic abnormalities (3, 4), advancing maternal age (5, 6), reproductive endocrine disease (7), and metabolic disorders (8). In addition, environmental pollutants including exposure to pesticides (9), insecticide (10), and bisphenol chemical compounds (11) have been shown to negatively affect reproductive health.

Diet, as a fundamental daily practice, plays a pivotal role in shaping overall health outcomes (12). It has been shown that populations adhering to diets rich in fruits, vegetables, fish, legumes, and whole grains exhibited significantly reduced rates of myocardial infarction (13, 14). In a more recent contribution, it has been reported that dietary supplementation with magnesium hydride (MgH_2_) conferred protective effects against chronic kidney disease induced by high-fat diet consumption (15). In contrast, a growing body of evidence indicates that unhealthy dietary patterns and a sedentary lifestyle contribute substantially to the onset and progression of chronic diseases (16) as well as reproductive disorders (17–19), and increased risk of severe non-alcoholic fatty liver disease and arterial hypertension (20, 21). Interestingly, sugar consumption has gradually increased in the Western world, and numerous studies showed its enrollmentassociation with several chronic diseases including type 2 diabetes, cardiovascular disease, obesity, non-alcoholic fatty liver disease, tooth decay, neurocognitive diseases, and chronic inflammatory disorders (22). Furthermore, there are studies that showed the roles of dietary sugar on ovarian dysfunction (23–25). Consequently, there has been a growing shift toward food products that contain non-sugar sweeteners (NSSs) instead of simple sugars (mono- and disaccharides) in order to reduce energy intake and prevent other diseases (26).

Sweeteners are broadly classified into two main categories. Nutritive sweeteners consist of natural and processed sugars that provide calories (energy) and are commonly used to sweeten foods and beverages (27). In contrast, non-nutritive sweeteners (NNS), also referred to as artificial sweeteners, are low- or non-caloric compounds used to impart sweetness without significantly contributing to energy intake. There are several types of NNS including aspartame, saccharin, stevia, and a plethora of sugar alcohols (polyols). Examples of polyols include xylitol, sorbitol, maltitol, erythritol, and lactitol (28).

Erythritol is a naturally occurring four-carbon polyol with approximately 75% of the sweetness of sucrose, low hygroscopicity, a characteristic cooling effect, and high crystallization capability (29). It received regulatory approval as a food additive in Japan in 1990 (30), and was later generally classified as Generally Recognized as Safe (GRAS) by the U.S. Food and Drug Administration (FDA) and the European Union Scientific Committee on Food (SCF) alongside aspartame and saccharin (31). Early studies have implied the potential benefits of erythritol, including its low insulin index and the fact that it does not significantly affect blood glucose levels, making it suitable for individuals with diabetes (32). Additionally, it does not contribute to dental caries and may even exert protective effects against tooth decay (30). In addition, some studies have also reported the antioxidant properties of erythritol and its potential to reduce blood glucose levels under diabetic conditions (33). However, despite its regulatory approval and reported benefits, several recent studies revealed the potential adverse effects of erythritol, including possible associations with cancer and cardiovascular disease (31).

Although erythritol is commonly used as a non-nutritive sweetener and is thought to be safe, nothing is known about how it can affect the physiology of female reproduction. New research indicates that dietary ingredients and artificial sweeteners may affect intracellular survival signaling pathways, redox balance, and endocrine homeostasis—all of which are essential for ovarian function. However, little is known about how ovarian architecture, follicular dynamics, and molecular regulatory networks are affected by erythritol exposure during crucial developmental windows, especially gestation. The goal of the current study was to methodically examine how exposure to gestational erythritol affects ovarian shape and function throughout generations. Our specific goals were to assess follicular growth patterns, ovarian histological changes, and markers linked to steroidogenesis such anti-Müllerian hormone (AMH) and Cyp19. To further clarify the molecular connections between erythritol exposure and ovarian dysfunction, we also looked at oxidative stress measures [malondialdehyde (MDA), superoxide dismutase (SOD), and glutathione (GSH)], autophagy-related markers (LC3 and Atg5), the apoptosis marker caspase-3, and the PI3K–p85 survival signaling pathway. This study aims to ascertain whether erythritol consumption causes ovarian stress, interferes with folliculogenesis, and modifies important survival and regulatory signaling pathways that preserve reproductive competence by combining structural, biochemical, and molecular analyses in female offspring of the first and second generations.

## 2 Materials and methods

### 2.1 Experimental animals

A total of 33 healthy adult female Wistar albino rats that weighed approximately 180–200 g were used in the present study. The rats used in this study were from Animal House, College of Science at King Saud University. Rats were housed separately in standard polypropylene mouse cages in a controlled environment for 14 days. Moreover, they were well ventilated and maintained under standard experimental conditions (temperature 23 °C, humidity 60%–70%, and a 12-h light/dark cycle), and the rats were given unrestricted access to food and water during the experimental period. All work with rat was done with the approval and under the supervision of the Scientific Research Ethics Committee (Reference No. KSU-SE-24-77) at King Saud University in Riyadh, Saudi Arabia. It was conducted following the established guidelines. All the experimental protocols adhered to and complied with the Animal Research: Reporting of *In Vivo* Experiments (ARRIVE) guidelines.

### 2.2 Study design

After acclimatization, 33 female rats (F0) were randomly tiered into three groups (*n* = 11), Group 1 (control), Group 2 (Dose 1), and Group 3 (Dose 2), and housed with untreated males in the ratio of 1:1. Then, the males were removed after 24 h, and successful mating was confirmed by the presence of a vaginal plug. After successful pregnancy, females received erythritol purchased in crystal form that was mixed in drinking water to make it suitable for oral ingestion in a dose-dependent manner:

Group 1 (control): Rats received only distilled water and a regular diet for 2 weeks.

Group 2 (Dose 1): Rats were given erythritol (0.4 g/kg/day) orally along with regular diet for 2 weeks.

Group 3 (Dose 2): Rats were given erythritol (4 g/kg/day) orally along with regular diet for 2 weeks.

Both Groups 2 and 3 were given erythritol on day 6 of gestation, which corresponds to the time of fetal implantation, and continued until gestation day (approximately 21–22 days). Body weight measurements of F0 rats in the whole group were taken daily for follow-up and then the average weight gain of the rat’s body weight was calculated. After delivery, the offspring that we acquired from F0 mothers that received erythritol treatment after parturition were named first generation (F1), whereas the control group was known as the first-generation control group (CF1). Moreover, body weight measurements of the newborns across the different groups and generations were obtained by calculating the average weight of the newborns. The sex ratio was determined by calculating the ratio of the number of females to the number of males. On week 4 (prepuberty), a portion of the treated female F1 and CF1 offspring (10 females from each group) were anesthetized and humanely sacrificed by transferring them individually into a glass jar and inhaling chloroform for blood and ovary sample collection (see Section 2.3).

After being allowed to reach sexual maturity, the surviving F1 and CF1 female rats were mated with healthy male rats. The body weight measurements of rats in the whole group were taken daily for follow-up. The F1 female rats treated with erythritol gave birth to a second generation of offspring (F2), whereas the control group rats produced a separate control subgroup for the second generation (CF2). Offspring weight was measured as well as the sex ratio. When offspring reached week 4 (prepuberty), portions of female rats were anesthetized and humanely sacrificed using the same method mentioned in Section 2.3.

### 2.3 Blood and tissue sample collection

When F1 and F2 offspring rats reached week 4 (prepuberty), they were anesthetized and humanely sacrificed by transferring individually into glass jars and inhaling chloroform. After weight measurement was obtained, blood sample was collected by cardiac puncture and transferred into blood test tubes to coagulate, and then centrifugation was performed at 5,000 rpm for 15 min at room temperature to separate the plasma and stored at –80°C for later use. At the same time, after blood collection, ovaries were quickly detached from oviducts and cleaned from surrounding adipose tissues, and both ovaries were weighted, labeled according to their origin groups, and then fixed as follows: some of the ovaries were fixed in 10% neutral buffer formalin (NBF) for histology and immunofluorescence (IF). Parts of ovaries were snap-frozen in liquid nitrogen and stored at –80 °C for later use to conduct oxidative stress and Western blot assays. Others were collected and stored in RNALater (Molequle-ON, New Zealand) at –80 °C, to perform real-time quantitative reverse transcription polymerase chain reaction (real-time qRT-PCR). Lastly, part of the ovaries were incubated in 2.5% glutaraldehyde for TEM assay. The ovary weight index was calculated as follows: average weight of the two ovaries divided by the weight of the corresponding female.

### 2.4 Histological preparation

Extracted ovaries were fixed in 10% NBF for 24 h, then all samples were dehydrated and embedded in paraffin wax blocks, after which they were sectioned (4 μm thick) by using the Rotary Microtome, RM 2245 (Leica, Germany) and stained with hematoxylin and eosin (H&E) for classic histological study according to the Mayer modified staining method (Bancroft & Gamble 2008) by using the Autostainer machine (Leica ST 5020, Lecia, Germany). For IF analysis, some blocks were cut into 3-μm-thick sections. Finally, follicular counting was determined for each group by using the method described in (34).

### 2.5 Immunofluorescence staining and confocal microscopy

Initially, sample slides were placed on a hotplate set at 60 °C for 2 h and then deparaffinized with xylene (2× 10 min) each. Tissue sections were rehydrated by ethanol (100% 2× 7 min, 95% 2× 7 min, 80% 7 min, 70% 7 min, and 50% 7 min) and samples were washed twice with distilled water and three times with phosphate buffer saline (1× PBS). After washing, slides were dried through placing them diagonally on a surface containing fine tissues to ensure the washing solution was completely removed. After drying, the slides were placed in a container filled with layers of fine tissues that were moistened with water to minimize the rate of evaporation of the solution that the tissue sections will be treated with in the next process. Following that, 0.1% Triton X-100 with 0.1% sodium citrate was added to the tissue sections. For staining, samples were incubated with blocking buffer [1% FBS (Capricorn Scientific) in PBS] at room temperature for 45 min to prevent non-specific background staining. Subsequently, the slides were placed into a humid box and were then incubated with Rabbit anti-LC3B (1:500 dilutions, Abcam) primary antibody. The humid container was covered carefully and kept overnight at 4 °C on a flat surface. The following day, slides were washed with 1× PBS (4× 5 min) each and then incubated with anti-rabbit Alexa Flour 488 (1:500 dilution, Abcam, USA) secondary antibody for 1 h at room temperature in the dark. After that, slides were washed with 1× PBS twice and with TE buffer and then incubated with Hoechst solution (1:15,000 dilution, Hoechst 33342, Life Technologies, USA). After the final incubation, the slides were washed with TE buffer, dried, and covered with coverslips beside adding nail polish to ensure that the edges were sealed tightly. The sections were observed and imaged for signal quantification using a spinning disk confocal microscope from Zeiss (10). The signal intensity for protein expression was analyzed by the Zen 3.1 service (ZEN lite) and quantified using the GraphPad Prism version 10 (GraphPad Software).

### 2.6 SDS-PAGE and Western blot analysis

For protein extraction, the ovarian tissues were homogenized with cold RIPA lysis buffer containing a protease inhibitor (Molequle-ON, New Zealand) in the homogenizer tube (GentleMACS™ M-Tubes, Miltenyi Biotec, Germany). The solution was centrifuged at 13,000 rpm for 15 min at 4 °C, then the supernatants were collected and transferred into a fresh tube and the protein concentration was determined using a Bradford test. Thereafter, 20 μg of protein was reduced in 2× Laemmli reducing buffer (Molequle-ON, New Zealand) and resolved on an SDS-PAGE gel (4–20% precast, Molequle-ON, New Zealand) using protein molecular weight standards (Molequle-ON, New Zealand). After protein separation, gels were then transferred onto polyvinylidene difluoride (PVDF) membranes (Molequle-ON, New Zealand) using wet blotting. After transfer, PVDF membranes were blocked in fetal bovine serum (FBS, Capricorn Scientific) blocking buffer for 1 h at room temperature, then incubated with primary antibodies (B-actin, Amh, LC3, Casp3, Cyp19, and PI3K–p85). The day after, membranes were washed (3× 10 min) with Tris-buffered saline with 0.1% Tween-20 (TBST) then incubated with horseradish peroxidase-linked secondary antibodies (1:1,000 dilution in FBS) for 1 h at room temperature; later, the membranes were rewashed with TBST (3× 10 min). Finally, the PVDF membranes were incubated in Western Substrate Plus (Molequle-ON, New Zealand) for a few minutes, and chemiluminescent signals were captured using a ChemiDoc MP Imaging System (Bio-Rad, USA).

### 2.7 Oxidative stress measurement

The level of lipid oxidation was assessed by measuring lipid peroxidation products as thiobarbituric acid reactive substances (TBARS), mainly MDA. Lipid peroxidation levels were determined spectrophotometrically following the method described in (35). Samples were heated with thiobarbituric acid (TBA) at low pH and the absorbance of the resulting pink chromogen was measured at 532 nm. Total GSH levels were quantified using the rapid colorimetric procedure as described in (36). In this procedure, a yellow color develops when 5,5′ dithiobis-2-nitrobenzoic acid is added to sulfhydryl compounds, allowing GSH concentration to be measured at 405 nm. SOD activity was evaluated according to the method of McCord and Fridovich (1969) (37), by following the inhibition of cytochrome c reduction rate via the superoxide radical, and observed at 550 nm.

### 2.8 Analysis of gene expression

Ovarian rat tissues were previously preserved in an RNALater stabilization reagent (Molequle-ON, New Zealand) placed at −80 °C, and the total RNA was isolated from ovarian tissues using QIAzol lysis Reagent (QIAGEN, USA). The quality and integrity of the isolated RNA were verified by measuring the 260/280-nm ratio using a NanoDrop 2000 (Thermo Scientific), then reverse-transcribed into cDNA through a High-Capacity cDNA Reverse Transcription kit (Applied Biosystems, Carlsbad, CA) according to the manufacturer’s instructions. Real-time PCR (RT-PCR) was performed in triplicate for each sample using Fast SYBER Green Master Mix (Applied Biosystems, USA) and a QuantStudio™ 7 Flex Real-Time PCR System machine (Applied Biosystems, Hercules, CA). The gene-specific primers are shown in **Supplementary Table 1**.

### 2.9 Measurement of erythritol by LC-MS

Plasma erythritol was measured as previously described (38). Briefly, erythritol was extracted from animal plasma samples using a protein precipitation method. Internal standard (D6-erythritol) dissolved in methanol (100 µL, 2 µM) and ice-cold methanol (800 µL) was added to plasma (30 µL). The mixture was vortexed and centrifuged at 20,000 × *g* at 4 °C for 15 min. The clear supernatant was transferred to a glass tube and evaporated to complete 12 dryness under a stream of nitrogen. The alcohol groups were then acetylated by adding 100 µL of 4-dimethylaminopyridine (DMAP, 1 mg/mL in pyridine) and 100 µL of acetic anhydride. The mixture was vortexed and heated at 80 °C for 45 min. After evaporation of the excess acetylation reagent under nitrogen, a liquid–liquid extraction step was performed. Ethyl acetate (2 mL) and an aqueous HCl solution (500 µL, 100 mM) were added, followed by mixing and centrifugation at 2,000 × *g* at 4 °C for 10 min. The organic layer was transferred to a new glass tube and evaporated to dryness under a constant stream of nitrogen. The dry residue was reconstituted in 100 µL of a water/methanol mixture (1:1, v/v) containing 5 mM ammonium formate. A 3-µL aliquot of the processed sample was injected onto a reversed-phase C18 column (50 mm × 2.1 mm, 2.6 µm; Phenomenex, Torrance, CA) at a flow rate of 0.4 mL/min using a gradient elution program. The mobile phases consisted of 0.125% formic acid and 10 mM ammonium formate in water (solvent A) and 0.125% formic acid with 10 mM ammonium formate in 95% acetonitrile/5% water (solvent B). Detection and quantification were performed using a Waters Xevo TQ-XS triple quadrupole mass spectrometer (**Table 1**).

**Table 1:** Plasma erythritol quantification in maternal and offspring by LC-MS across two Generations.

Group	F0 Mother (G Day 15)]	F1 Offspring (Day 28)	F1 Mother (G Day 15)]	F2 Offspring (Day 28)
**Control**	5.48±0.00	6.98±0.801	5.30±0.00	6.44±1.890
**Dose-1^a^**	20.12±0.00	6.75±1.274	7.82±0.00	6.61±1.715
**Dose-2^b^**	4103.01±0.00	6.82±0.824	5.51±0.00	5.21±0.788

^(a)^ Erythritol (0.4 g/kg/day), ^(b)^ Erythritol (4 g/kg/day), F0: baseline Mother, F1: First-generation, F2: Second-generation, (G Day 15): gestational day 15 (pregnant mother), (Day 28) = pre-pubertal day 28 (offspring age).

### 2.10 Statistical analysis

Statistical analyses were conducted using GraphPad Prism version 10 (GraphPad Software version 10.2, San Diego, CA, USA) to compare gene and protein expression levels and the intensity quantification in IF assay between the control and treatment groups. One-way analysis of variance (ANOVA) was conducted, as well as Dunnett’s and Tukey’s multiple comparison tests for statistical analysis. The results were expressed as the mean ± standard deviation (SD). *p*-values less than 0.05 were considered statistically significant.

### 2.11 Ethical approval

This study was approved by the Scientific Research Ethics Committee (REC) at King Saud University, Riyadh, Saudi Arabia (Reference No. KSU-SE-24-77) and carried out in accordance with the approved guidelines.

## 3 Results

### 3.1 Effect of erythritol on offspring body weight and ovarian index

**Figure 1:**
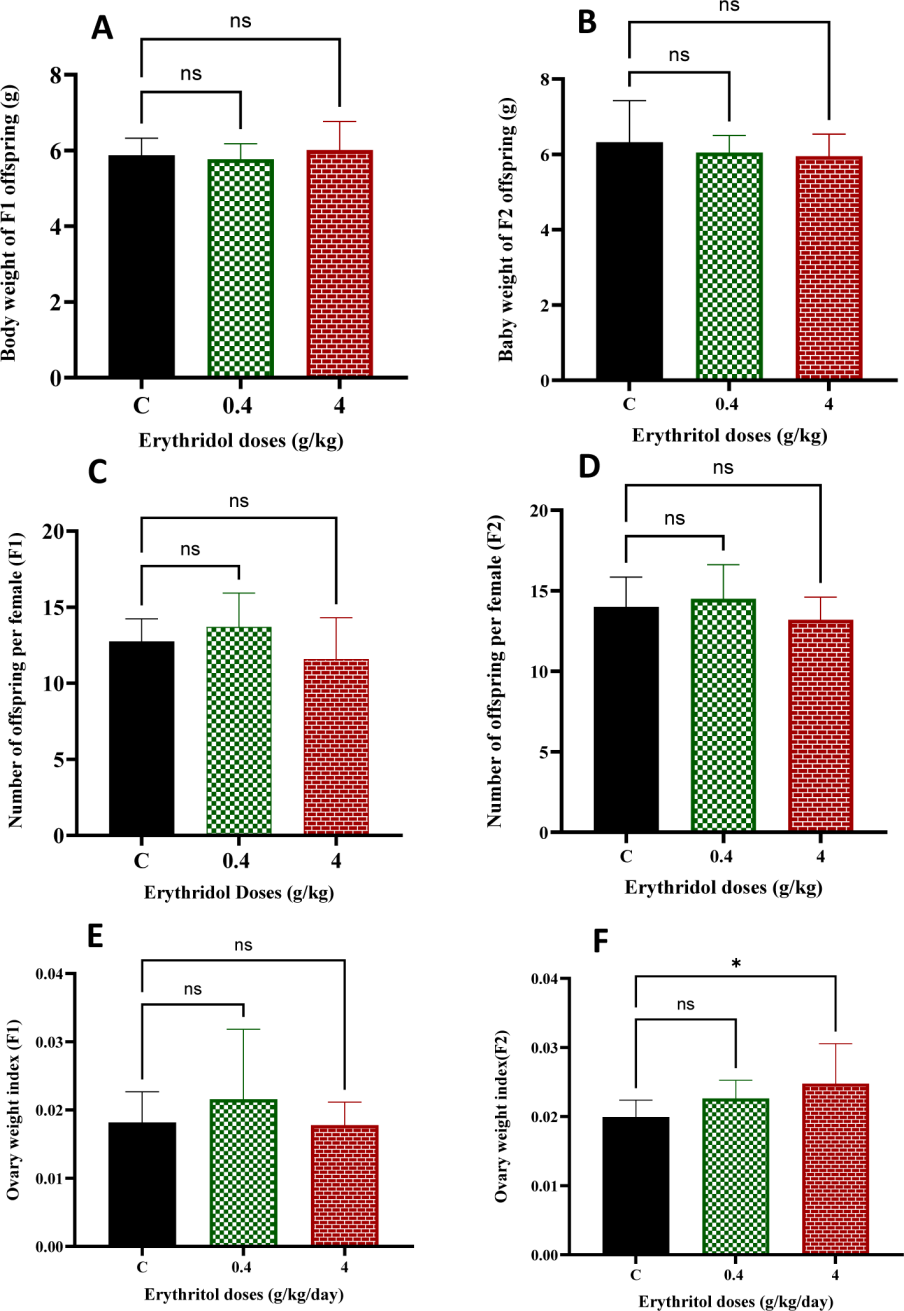
Body weight, the number of offspring, and ovarian indices of the F1 and F2 generations of offspring treatment groups were compared with those of the control group. In both the first and second generations, the body weight of offspring were not significant between treated groups and control groups **(A, B)**. Similarly, the number of offspring in both generations was not significantly different among the whole group **(C, D)**. Ovarian indices of the F1 generation in low and high doses presented not significant differences compared with the control group **(E)**. However, in the second generation, female rats in the high-dose erythritol treatment group presented a significant increase in the ovarian index compared with that in the control group **(F)**. Statistical significance was considered for all *p*-values. *p ≤ 0.05, ns, non-significant.

Body weight and ovarian index in prepubertal progeny are significant indications of systemic growth and the early development of reproductive organs. Body weight was assessed in prepubertal female progeny across the F1 and F2 generations. No statistically significant differences were seen between the erythritol-treated groups and the control groups in the F1 offspring (**Figure 1A**). The body weights of F2 offspring did not substantially differ from their corresponding controls (**Figure 1B**). The evaluation of fertility outcomes, measured by offspring quantity, indicated no significant differences among low-dose (0.4 g/kg) and high-dose (4 g/kg) erythritol groups compared to controls in both generations (**Figures 1C,D**). The ovarian index analysis revealed no significant alterations in F1 females across both treatment groups as compared to controls (**Figure 1E**). F2 females subjected to the elevated dosage of erythritol demonstrated a statistically significant enhancement in ovarian index relative to controls (*p* < 0.05) (**Figure 1F**). Although erythritol did not affect overall growth or fertility rates, the elevated ovarian index in F2 high-dose progeny indicates possible generational changes in ovarian development or structural reconfiguration.

### 3.2 Effect of erythritol on the number of follicles growing

**Figure 2:**
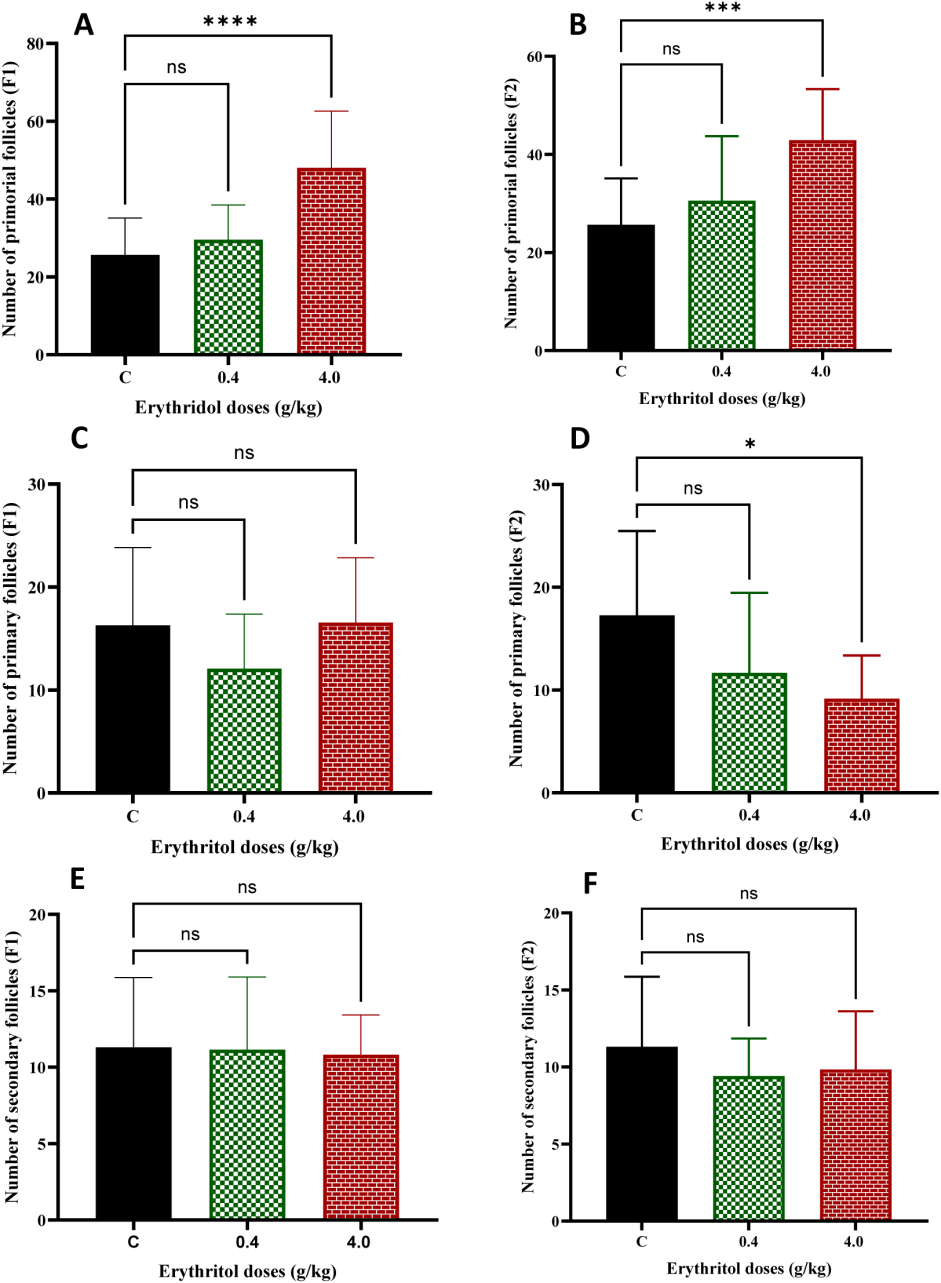
Primordial follicles **(A, B)**, primary follicles **(C, D)**, and secondary follicles **(E, F)** in F1 and F2 generations. *p ≤ 0.05, ***p ≤ 0.001, ****p ≤ 0.0001, ns, non-significant.

Follicular development advances from primordial to primary and secondary stages, with changes in these populations indicating disturbances in follicle recruitment and maturation dynamics. The effect of erythritol on ovarian folliculogenesis was evaluated in prepubertal F1 and F2 progeny. High-dose erythritol exposure (4.0 g/kg) markedly elevated the quantity of primordial follicles in both generations relative to controls, whereas low-dose therapy (0.4 g/kg) did not have a meaningful effect (**Figures 2A, B**). In the F1 generation, neither low nor high doses significantly affected the quantity of primary follicles. In the F2 generation, high-dose erythritol led to a slight yet significant decrease in primary follicle counts relative to controls (**Figures 2C, D**). No notable discrepancies were detected in secondary follicle counts between the treatment and control groups in both generations (**Figures 2E, F**). The data indicate that high-dose erythritol may modify early follicular pool dynamics and recruitment patterns, especially over generations, thereby disturbing the equilibrium between follicle activation and maturation.

**Figure 3:**
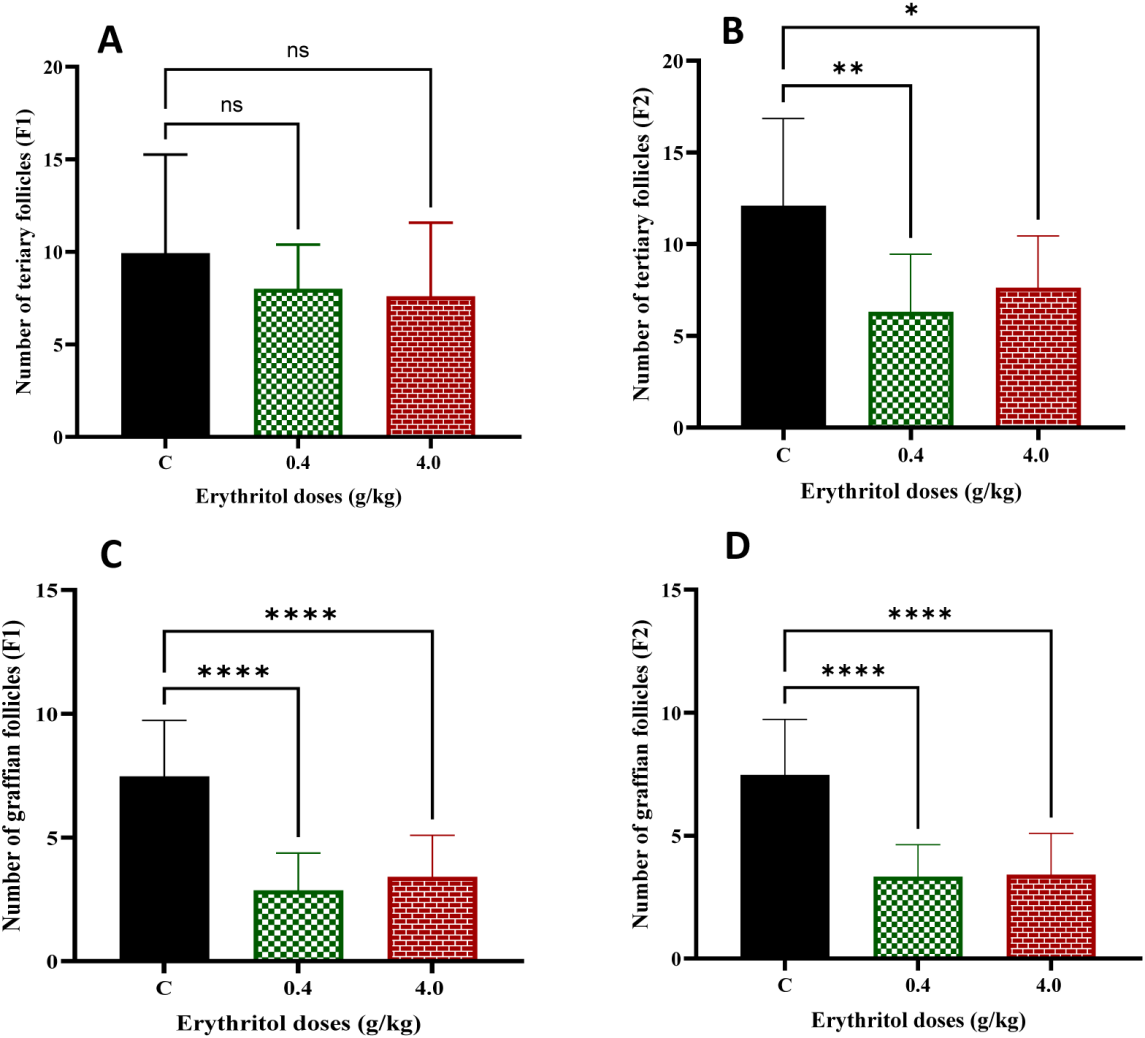
Number of tertiary follicles **(A**, **B)**. Graafian follicles **(C, D)** in F1 and F2 generations. *p ≤ 0.05, **p ≤ 0.01,****p ≤ 0.0001, ns, non-significant.

Tertiary and Graafian follicles represent advanced phases of follicular maturation. In the F1 generation, the quantities of tertiary follicles exhibited no significant variations between the control and erythritol-treated groups. In the F2 generation, both low- and high-dose treatments led to a substantial decrease in tertiary follicles compared to controls (**Figures 3A, B**). Graafian follicles were markedly reduced in both low- and high-dose erythritol-treated groups in the F1 and F2 generations compared to controls (**Figures 3C, D**). The findings imply that erythritol exposure hinders follicle development to the pre-ovulatory stage and modifies ovulatory-related structures over generations, indicating disturbed follicular progression and ovarian functional remodeling.

### 3.3 Effect of erythritol on ovarian histology

**Figure 4:**
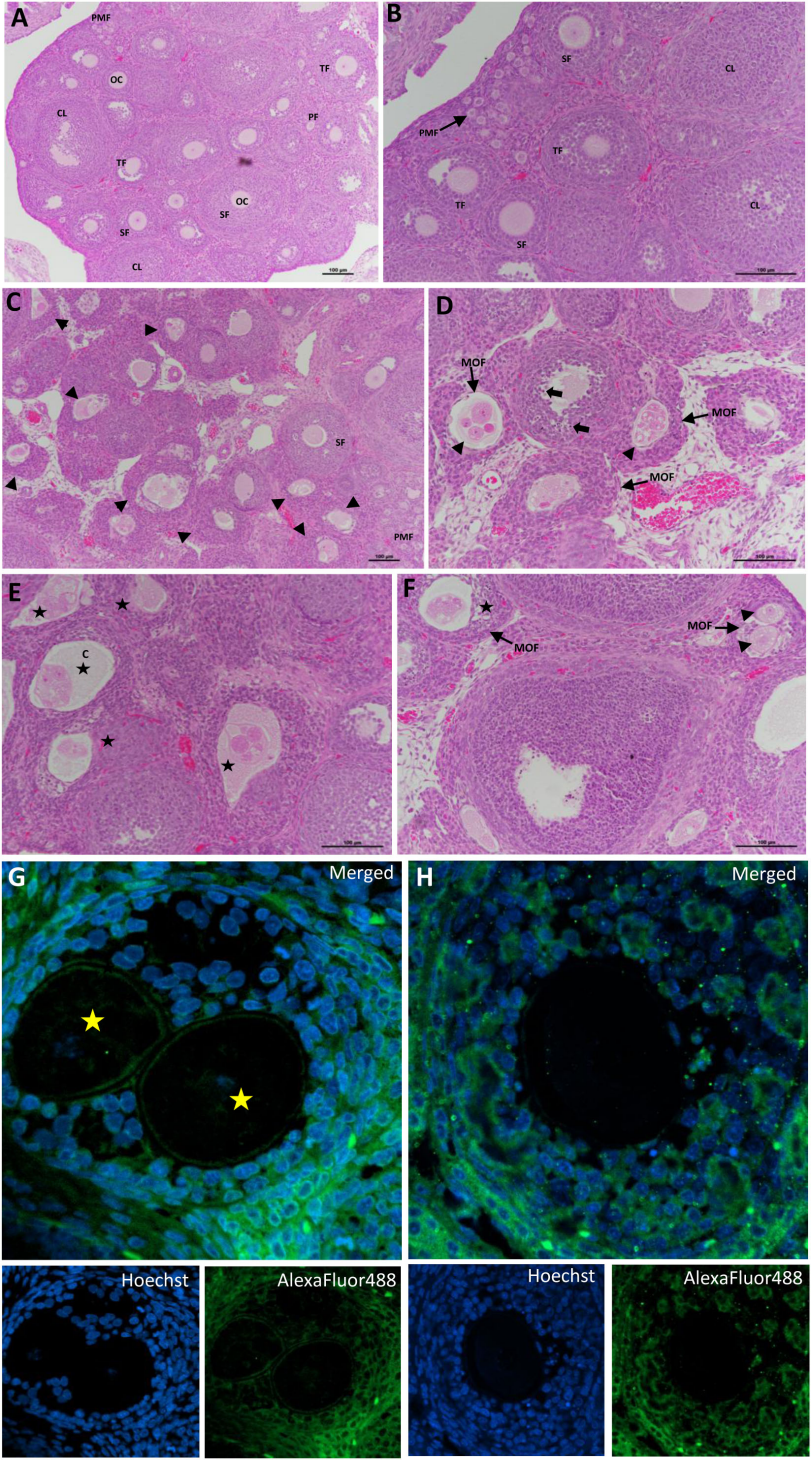
Photomicrographs of H&E-stained ovarian sections. Panels **(A)** and **(B)** illustrate the control group, showing a normal ovarian histological structure containing growing follicles. Photomicrographs of erythritol-treated ovaries **(C–F)** showed an altered structure in most of the growing follicles (arrowhead); a notable characteristic of these ovaries was the increase and presence of degenerating follicles that include abnormal oocytes, multi-oocyte formation (arrowhead), and elevated granulosa cells pyknotic nuclei (arrow); this finding was obtained with both doses. **(G, H)** An immunofluorescence of erythritol-treated ovaries revealed abnormal follicles that have two oocytes and oocyte vacuolization. DF, degenerative follicle; GC, granulosa cell; OC, oocyte; PMF, primordial follicle; PM, primary follicle; SF, secondary follicle; TF, tertiary follicle; GF, Graafian follicle; CL, corpus luteum; C, cysts.

Histological assessment of ovarian tissue offers direct evidence of follicular integrity, cellular organization, and structural indications of reproductive health. Histological analysis revealed normal ovarian structure in the control group, featuring well-organized follicles at various developmental stages (**Figures 4A, B**). The groups treated with erythritol displayed significant structural anomalies. In the F1 generation, ovaries exhibited modified follicular architecture, multi-oocyte follicles, multinucleated oocytes, and an elevated number of granulosa cells with pyknotic nuclei (**Figures 4C, D**). Significant modifications were noted in the F2 generation, encompassing heightened cyst development, deteriorating follicles, fragmented or numerous oocytes, and multinucleated oocytes (**Figures 4E, F**). IF analysis additionally validated the presence of aberrant follicles containing two oocytes and significant vacuolization surrounding the oocytes in erythritol-treated ovaries (**Figures 4G, H**). The structural abnormalities suggest that erythritol impairs normal follicular organization and cellular integrity, resulting in more pronounced transgenerational consequences that could jeopardize oocyte quality and ovarian functionality.

### 3.4 Erythritol induced oxidative stress in rat ovaries

**Figure 5:**
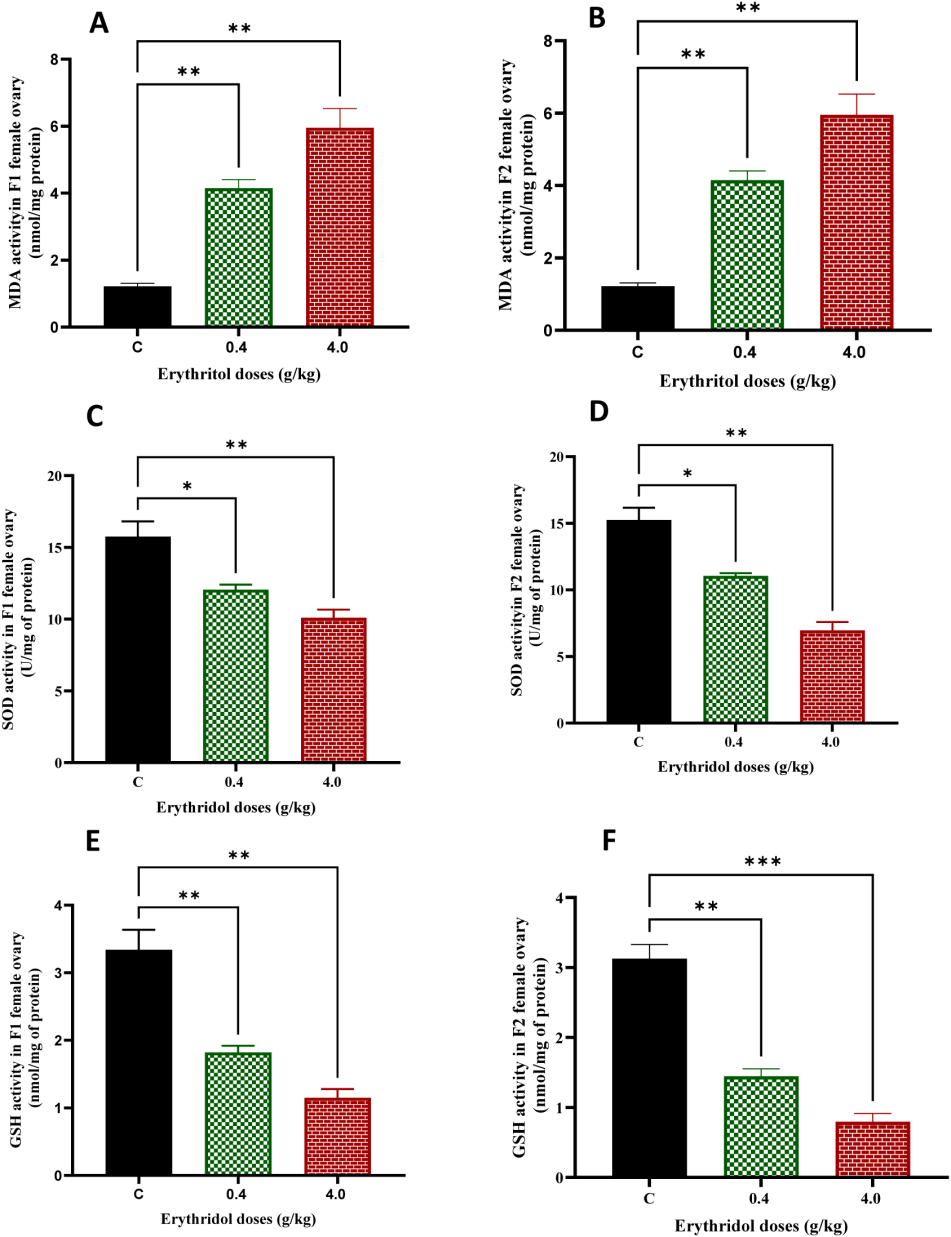
Erythritol treatment-induced oxidative stress. Pregnant female rats were exposed to 0.4 and 4.0 g of erythritol/kg body weight from day 6 of gestation until delivery, and the level of MDA **(A, B)**, SOD **(C, D)**, and GSH **(E, F)** in female offspring was then determined in the F1 and F2 generations during the prepubertal stage. *p ≤ 0.05, **p ≤ 0.01, ***p ≤ 0.001.

Oxidative stress significantly contributes to ovarian dysfunction by disturbing redox equilibrium, destroying cellular structures, and hindering follicular growth. The oxidative stress status was evaluated by quantifying the lipid peroxidation marker MDA and the antioxidant defense enzymes SOD and reduced GSH. MDA levels were markedly increased in both F1 and F2 progeny subjected to erythritol in a dose-dependent fashion compared to controls (*p* < 0.05) (**Figures 5A, B**). Conversely, antioxidant defense markers exhibited substantial declines, with both SOD activity and GSH levels significantly diminished in erythritol-treated groups across the F1 and F2 generations (**Figures 5C–F**). These alterations consistently signify redox imbalance in ovarian tissue subsequent to erythritol treatment. The findings indicate that erythritol causes enduring oxidative stress in the ovary, and the propagation of redox imbalance to the subsequent generation may lead to transgenerational ovarian dysfunction and compromised follicular integrity.

### 3.5 Impact of erythritol on the folliculogenesis and steroidogenesis-related markers

**Figure 6:**
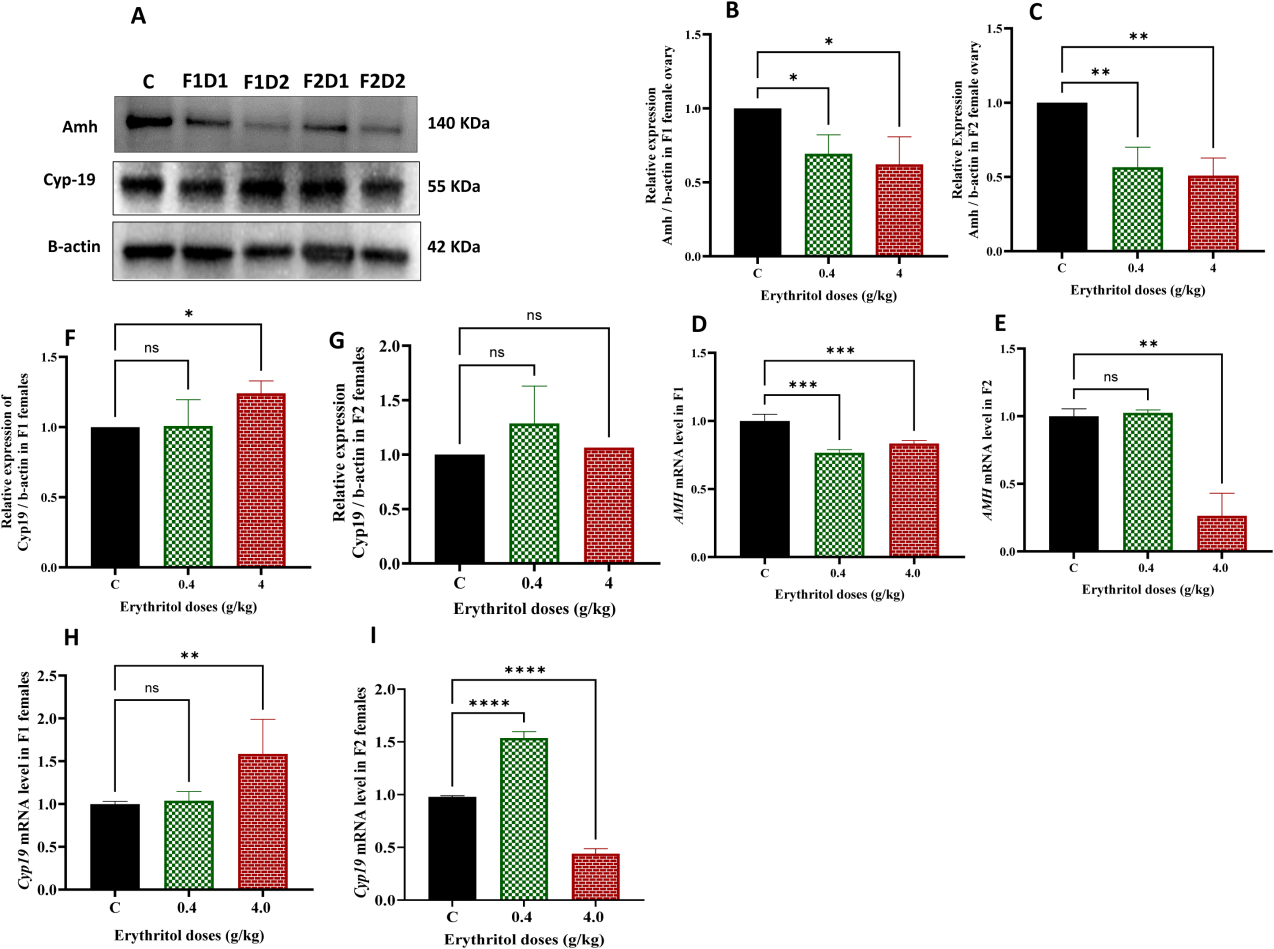
Effect of erythritol exposure on ovarian function markers in rat ovaries. **(A–G)** Representative immunoblot images showed the protein expression levels of AMH and Cyp19 in the ovaries of F1 and F2 generations. **(D–I)** Representative RT-PCR analysis of gene expression related to folliculogenesis and steroidogenesis in ovarian tissues of rats across different treatment groups, compared to the control group across two generations. F1D1, Dose 1 first generation; F1D2, Dose 2 first generation; F2D1, Dose 1 second generation; and F2D2, Dose 2 second generation. *p ≤ 0.05, **p ≤ 0.01, ***p ≤ 0.001, ****p ≤ 0.0001, ns, non-significant.

AMH governs follicle recruitment and indicates ovarian reserve, while Cyp19 (aromatase) is crucial for estrogen synthesis and proper steroidogenic activity. The molecular effects of erythritol on follicular development and steroidogenesis were assessed by analyzing the protein and mRNA expression levels of Amh and Cyp19 in F1 and F2 ovaries (**Figure 6**). Western blot analysis revealed a dose-dependent decrease in AMH protein expression in erythritol-treated groups relative to controls over both generations (**Figures 6A–C**). Amh mRNA expression was consistently and significantly reduced in both low- and high-dose groups in F1, whereas in F2, considerable downregulation occurred solely in the high-dose group (**Figures 6D, E**).

Conversely, Cyp19 protein expression was markedly elevated in the high-dose (D2) F1 group relative to controls, but no significant alterations were observed in F2 protein levels (**Figures 6A, F, G**). Nonetheless, Cyp19 mRNA expression was significantly diminished in F2 females subjected to the elevated dosage of erythritol (**Figures 6H, I**). The findings imply that erythritol interferes with essential regulators of folliculogenesis and steroidogenesis, indicating compromised ovarian reserve signaling and modified estrogen production, especially under situations of high-dose and transgenerational exposure.

### 3.6 Erythritol inhibited autophagy mechanism probably through epigenetic modification

**Figure 7:**
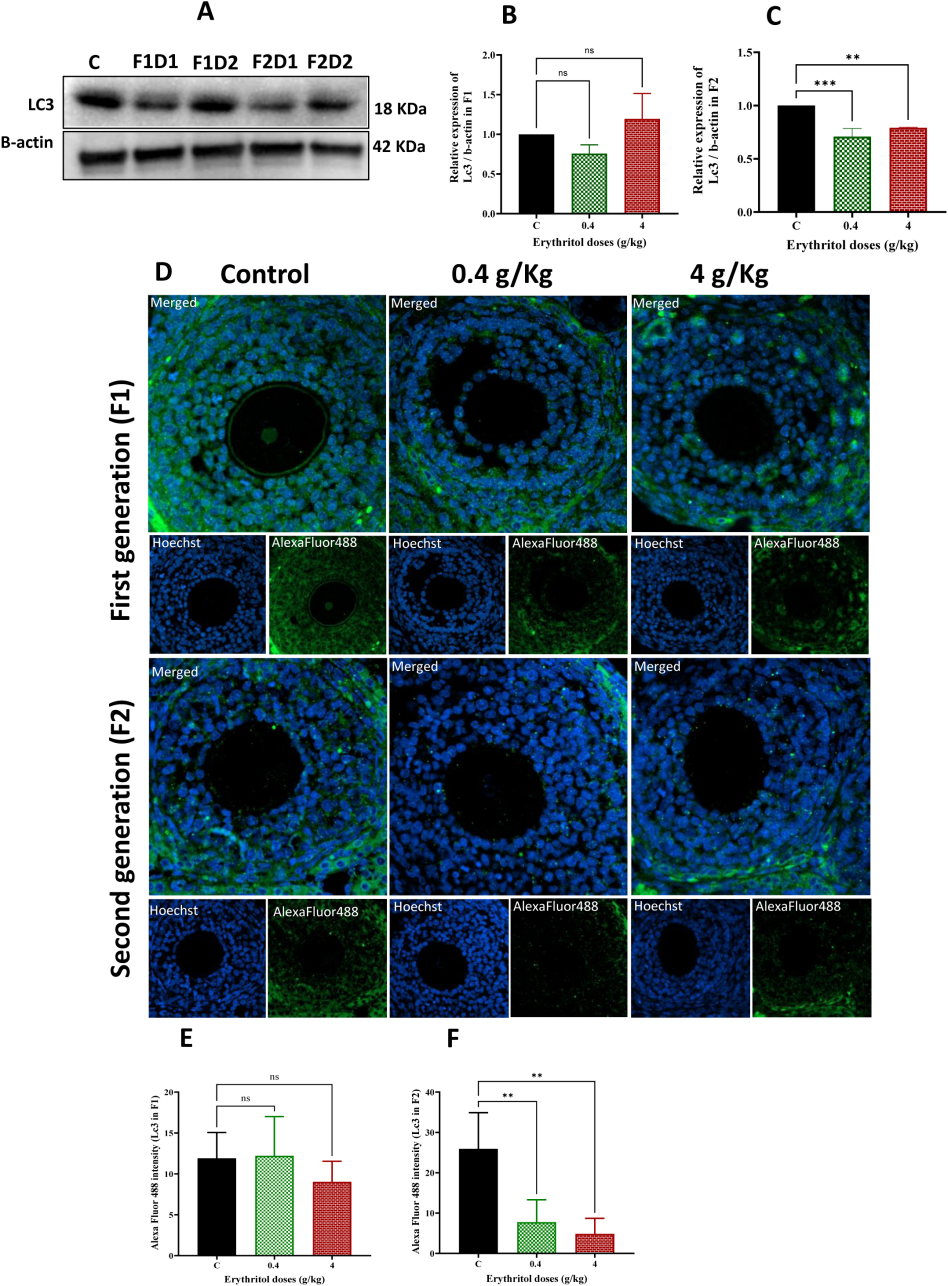
Autophagy in ovarian tissues was evaluated by Western blotting **(A–C)** and immunofluorescence staining with assessment of the relative fluorescence intensity of LC-3 **(D–F)** in the control and exposed groups. F1D1, Dose 1 first generation; F1D2, Dose 2 first generation; F2D1, Dose 1 second generation; and F2D2, Dose 2 second generation. **p ≤ 0.01, ***p ≤ 0.001, ns, non-significant.

**Figure 8:**
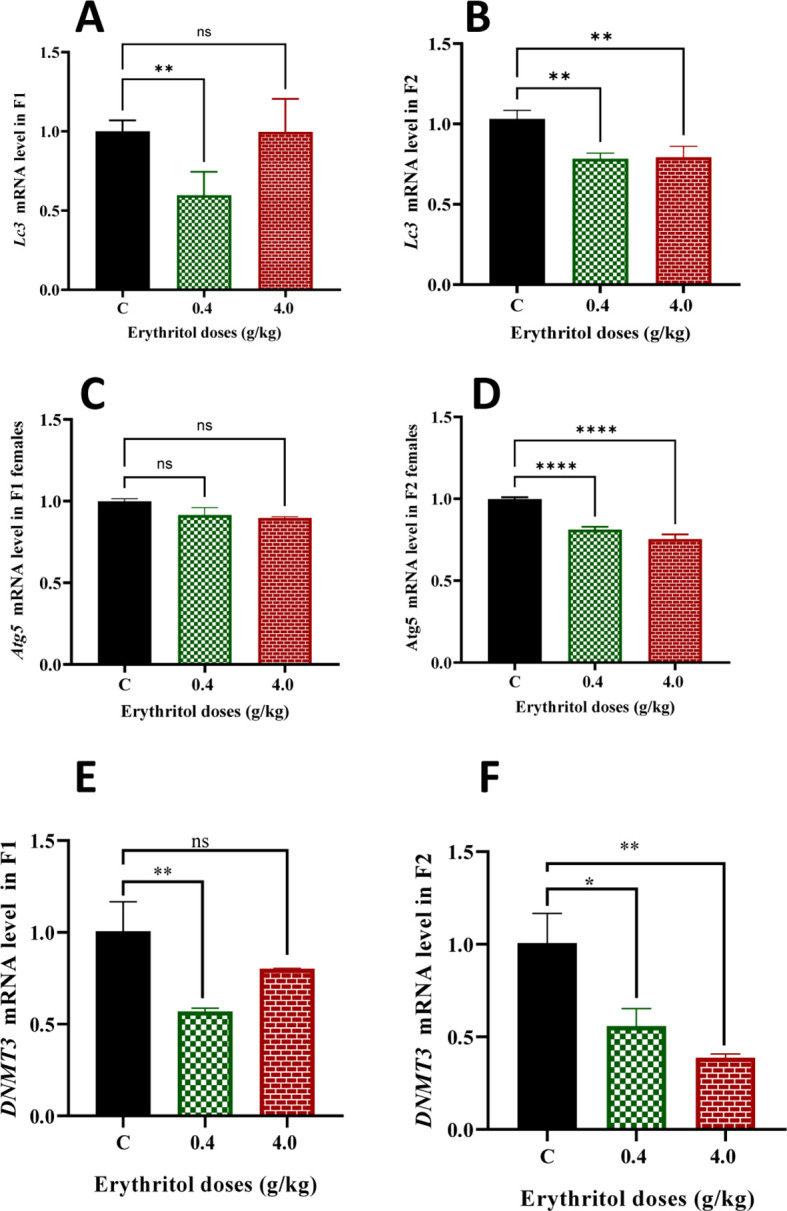
The mRNA levels of the *Lc3* and *Atg5* genes in female rats exposed to erythritol compared with those in the control rats were determined by RT-PCR **(A–D)**. The mRNA levels of the *Dnmt* gene in female rats exposed to erythritol compared with those in the control rats were determined in both generations by RT-PCR **(E, F)**. **p ≤ 0.01, ****p ≤ 0.0001, ns, non-significant.

Autophagy is crucial for sustaining ovarian homeostasis by safeguarding granulosa cell viability, mitochondrial integrity, and oocyte quality. To ascertain the involvement of autophagy in erythritol-induced ovarian dysfunction, the expressions of LC3 and Atg5 were evaluated using Western blotting, IF, and RT-PCR (**Figure 7**). In the F1 generation, LC3 protein levels exhibited no significant variations between the treatment groups (D1 and D2) and the controls (**Figures 7A–C**). IF analysis consistently demonstrated no significant alterations in LC3 fluorescence intensity in F1 ovaries (**Figures 7D, E**). In the F2 generation, LC3 protein expression was markedly diminished in both low- and high-dose groups relative to controls (**Figures 7A–C**), and IF corroborated the reduction in LC3 intensity (**Figures 7D, F**). At the transcript level, Lc3 mRNA was dramatically diminished in F1 D1 offspring, but both treatment groups in F2 demonstrated pronounced downregulation (**Figures 8A, B**). Likewise, Atg5 mRNA levels remained constant in F1 but exhibited a significant reduction in both F2 treatment groups (**Figures 8C, D**). The data indicate that erythritol mostly inhibits autophagy signaling in the second generation, potentially compromising cellular quality control mechanisms and leading to transgenerational ovarian dysfunction.

Concerning the epigenetic modification marker, the mRNA level of *Dnmt3* gene in the F1 generation was reduced in the low-dose group. In contrast, the gene expression remained unchanged in the high-dose (4 g/kg) group compared to the control (**Figure 8E**). In the F2 generation, *Dnmt3* mRNA expression was significantly reduced collectively in the low- and high-dose erythritol group relative to the control group (**Figure 8F**).

### 3.7 Effect of erythritol on the expression of apoptosis and PI3k signaling pathway markers

**Figure 9:**
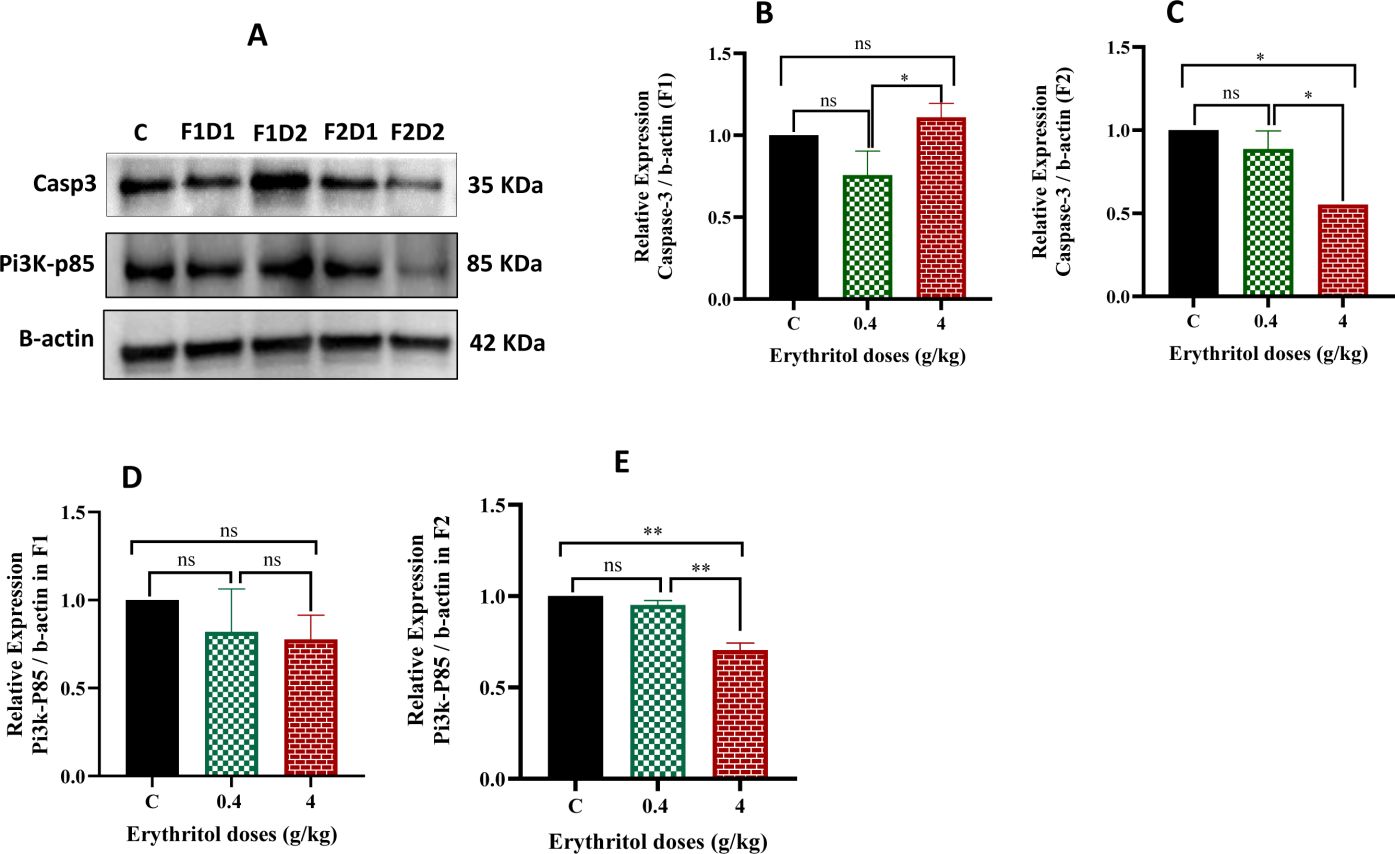
Effect of erythritol on the expression of apoptosis markers at the protein level in the rat ovaries of the first and second generations **(A–E)**. The protein expression levels of active caspase-3 and PI3K–p85 were examined by Western blotting. F1D1, Dose 1 first generation; F1D2, Dose 2 first generation; F2D1, Dose 1 second generation; and F2D2, Dose 2 second generation.*p ≤ 0.05, **p ≤ 0.01, ns, non-significant.

This study examines the impact of erythritol exposure on apoptotic signaling and the PI3K survival pathway in ovarian tissue by evaluating the protein expression levels of active caspase-3 and PI3K–p85 using Western blotting in F1 and F2 female offspring (**Figures 9A–E**). In the F1 generation, caspase-3 protein expression remained unchanged in the low-dose (0.4 g/kg) group compared to the control, but the high-dose (4 g/kg) group had a minor although statistically significant increase in relation to the low-dose group (**Figure 9B**). Nonetheless, no substantial change was detected between the control and high-dose groups, suggesting a restricted pro-apoptotic effect in F1 ovaries.

Conversely, in the F2 generation, caspase-3 expression was markedly diminished in the high-dose group relative to the control (**Figure 9C**), indicating a disrupted apoptotic equilibrium in the second-generation progeny. Concerning the PI3K signaling pathway, the expression of the PI3K–p85 protein in the F1 generation did not exhibit significant differences across treatment groups compared to the control (**Figure 9D**). In the F2 generation, PI3K–p85 expression was markedly reduced in the high-dose erythritol group relative to both the control and low-dose groups (**Figure 9E**). This lowering signifies the inhibition of PI3K signaling in second-generation ovarian tissue after high-dose exposure.

## 4 Discussion

This study presents comprehensive morphological, biochemical, and molecular evidence indicating that prenatal and early life exposure to erythritol disrupts ovarian homeostasis throughout two generations. While erythritol has traditionally been regarded as a low-toxicity sugar alcohol according to subchronic and reproductive toxicity studies, recent findings from non-nutritive sweetener research indicate that metabolic context, redox signaling, and developmental phases can significantly alter biological effects (39). This study identifies distinct ovarian-level alterations in follicle development, histological integrity, oxidative stress, autophagy markers, and PI3K signaling, indicating that ovarian molecular endpoints may exhibit greater sensitivity than overt reproductive outcomes, especially during early prepubertal stages.

At the systemic level, the body weights and offspring numbers of F1 and F2 progeny did not significantly differ from the controls (**Figures 1A–D**), indicating that erythritol exposure did not result in overt growth restriction or substantial fertility failure; however, variations in these general endpoints should not be construed as an absence of reproductive toxicity. Developmental reproductive toxicology often demonstrates that changes in the endocrine system and ovarian microenvironment can result in subclinical ovarian aging phenotypes, compromised follicle quality, or signaling abnormalities that become evident prior to the observable loss in fertility (40). The ovarian index in F2 high-dose children rose (**Figure 1F**), indicating a generational divergence in ovarian response.

In our study, the quantitative follicle count analyses demonstrate that erythritol exerts generation-specific effects on follicular development across both the first and second generations. The high-dose group markedly elevated primordial follicle reservoir in F1 (**Figures 2A, B**). While the numbers of primary, secondary, and tertiary follicles showed no significant differences in the erythritol-treated F1 groups relative to controls, Graafian follicle counts were significantly reduced across both doses (**Figures 3C, D**). These findings suggest that F1 offspring females exposed to erythritol *in utero* exhibit a disruption in the follicular recruitment-to-maturation continuum, potentially reflecting aberrant follicular arrest or elevated atresia of advanced-stage follicles (41). Notably, despite the absence of direct *in utero* erythritol exposure in F2 offspring females, whose only exposure was indirect through their exposed mothers, both tertiary and Graafian follicle counts were significantly reduced in their ovaries. These findings indicate that erythritol exposure induces transgenerational effects, culminating in ovarian developmental toxicity, a diminished reproductive lifespan, and accelerated ovarian aging (9). Similar findings have been observed for other dietary and chemical exposures that affect oxidative stress and survival pathways (19, 42, 43). For instance, artificial sweeteners like aspartame have been documented to adversely affect ovarian follicle reserve and steroidogenic function in animal settings, with researchers highlighting oxidative stress and impaired ovarian signaling as possible reasons (44). These findings correspond with the current results that follicle development is selectively compromised despite the preservation of body weight and overall fertility measures.

The histopathological study enhances the interpretation of follicle count by revealing qualitative ovarian damage. Indeed, ovaries from F1 and F2 treated females displayed modified follicle architecture, multi-oocyte follicles, granulosa cell pyknotic nuclei, and multinucleated oocytes in F1 (**Figures 4C, D**), along with an increase in cysts, degenerating follicles, and fragmented or multiple oocyte production in F2 (**Figures 4E, F**). IF images additionally reveal aberrant follicles containing two oocytes and exhibiting vacuolization (**Figures 4G, H**). These characteristics indicate compromised oocyte-follicular stability and aberrant oocyte developmental programming (34, 45). Multi-oocyte follicles and granulosa cells are indicative of impaired follicle assembly or disrupted communication within the follicular niche, processes susceptible to oxidative stress, endocrine dysregulation, and altered growth factor signaling (46). The observation of anomalies at both doses, along with significant degenerative results in F2, reinforces the notion of developmental reprogramming and cumulative stress signaling over generations (9, 34).

Biochemical analyses reveal significant redox imbalance in ovarian tissue (47). MDA exhibited a dose-dependent increase in both generations, but antioxidant defenses SOD and GSH demonstrated a decline. Oxidative stress is an established catalyst of follicular atresia, granulosa cell dysfunction, mitochondrial impairment, and expedited ovarian aging (48). Literature on ovarian aging suggests that oxidative stress induces apoptosis, mitochondrial malfunction, and signaling dysregulation in the ovary, and that reestablishing redox balance may safeguard follicular survival pathways, including PI3K–AKT–FoxO signaling (49). Redox imbalance offers a biologically coherent rationale for the study’s integrated findings: oxidative stress can compromise granulosa cell function, diminish oocyte quality, inhibit protective autophagy when chronic or severe, and redirect signaling away from survival and maturation, resulting in the combined phenotypes.

AMH has emerged as one of the most robust biomarkers of ovarian reserve due to its early age-related decline and relative independence from menstrual cycle phase or hormonal contraception (50). AMH levels progressively decrease with advancing age in parallel with the depletion of the ovarian follicle pool, becoming undetectable after menopause (51). Results of the current research identified a dose-dependent decrease in AMH protein expression across both generations, corroborated by diminished *Amh* mRNA and proteins levels. AMH functionally inhibits primordial follicle recruitment and indicates the health of growing follicles (52). A diminished AMH level is frequently regarded as an indication of poor granulosa cell functionality and reduced follicular competence (53). The AMH results align with the follicle account and histological evidence of aberrant follicles found in ovaries from treated offspring females. Previous research on non-nutritive sweeteners, especially aspartame, indicates ovarian dysfunction and altered reproductive hormone feedback (54), reinforcing the hypothesis that dietary sweeteners may affect ovarian endocrine biology and follicle health in vulnerable situations. The absence of significant differences in *Cyp19* expression across both treatment groups and generations suggests that the transgenerational effects of erythritol are mediated through mechanisms independent of the FSH–aromatase axis (55).

Autophagy is crucial for preserving granulosa cell homeostasis, oocyte quality, mitochondrial integrity, and tolerance to metabolic stress (56, 57). In the present study, LC3 protein levels showed no significant alterations in F1 offspring; however, a marked reduction was observed in F2 offspring at both doses. These findings were further corroborated by IF, which confirmed significantly reduced LC3 intensity in F2. Consistent with these protein-level changes, transcriptional analysis revealed considerable downregulation of *Lc3* and *Atg5* in F2 offspring. This pattern indicates low impact on F1 protein endpoints but significant reduction in F2 offspring. Given that second-generation offspring were not directly exposed to erythritol, these findings strongly implicate a transgenerational susceptibility mechanism, suggesting that F2 ovaries may possess a diminished capacity to mount adequate responses against oxidative and metabolic stress. The transgenerational nature of this effect may be attributable to epigenetic reprogramming, as our analysis revealed a significant reduction in global DNA methylation levels in F2 offspring across both treatment groups relative to controls. These observations are consistent with a growing body of literature demonstrating that early-life conditions can durably alter the methylation status of candidate genes in brain regions critically involved in stress signaling and transmission (58). Indeed, epigenetic imprinting is particularly active during germline cell formation and embryonic development, periods during which DNA methylation plays a pivotal role in reprogramming cells to establish future physiological functions (59, 60).

PI3K signaling is crucial for follicle activation, granulosa cell survival, and the preservation of ovarian reserve (61). In this study, the PI3K–p85 protein exhibited no significant changes in F1 (**Figure 9D**), but demonstrated a substantial reduction in F2 high-dose offspring compared to both control and low-dose groups (**Figure 9E**). This result is significant as disruption of the PI3K pathway may result in aberrant follicle activation dynamics, hindered maturation, and heightened follicular degeneration (61). The F2-specific inhibition of PI3K–p85 corresponds with the more pronounced structural disruption in F2, diminished tertiary follicles, and inhibited autophagy. The correlation between PI3K disruption, oxidative stress, and autophagy is corroborated by previous ovarian toxicological studies on insecticide exposure (62), which have documented ovarian damage linked to oxidative stress and alterations in the PI3K/AKT/mTOR pathway (63), thereby connecting redox imbalance to the impairment of survival signaling. The study references a mechanistic framework based on previous research with allethrin exposure and PI3K/AKT/mTOR signaling in developing rat ovaries (10). Reviews of premature ovarian insufficiency indicate that antioxidant interventions can partially restore ovarian function by activating PI3K–AKT–FoxO pathways and enhancing antioxidant defenses, underscoring that the suppression of the PI3K pathway may result from oxidative stress (64).

The results for caspase-3 were inconsistent between generations and limited; nevertheless, in the F2 high-dose group, caspase-3 expression was diminished. This result indicates a dysregulated apoptotic balance rather than a direct stimulation of apoptosis, presumably reflecting altered cell turnover, compensatory responses, or temporal implications related to the prepubertal collection window. In developmental circumstances, diminished caspase-3 may be associated with irregular follicular remodeling or the inappropriate retention of faulty cells, thereby contributing to the observed atypical follicle morphologies (65). The data indicate numerous quality-control systems in the ovary, including redox defense, autophagy, and survival signaling, particularly when analyzed in conjunction with repressed autophagy and diminished PI3K survival signaling.

A prevailing theme is that F2 ovaries exhibit more pronounced molecular abnormalities than F1 regarding autophagy and PI3K–p85, despite the presence of oxidative stress in both generations. This discovery aligns with developmental programming, wherein gestational experiences influence fetal ovarian growth and later ovarian susceptibility to stress (66). This work did not directly assess epigenetic endpoints; however, the pattern supports the concept that erythritol exposure during crucial periods may modify ovarian developmental trajectories, endocrine responsiveness, or redox set points, hence increasing sensitivity in subsequent generations (67). This transgenerational signal offers a mechanistic framework for reconciling previous erythritol reproductive research (68), which concentrated on overall fertility and teratogenicity, with the current study’s ovarian molecular observations. Conventional endpoints may overlook tissue-specific aging-related or subfertility abnormalities that necessitate histology and genetic analysis for identification.

While the emphasis is on reproductive biology, it is important to acknowledge that erythritol has lately been linked to thrombosis-related cardiovascular risk in observational and mechanistic investigations, leading to demands for additional safety assessment (69). The data do not demonstrate direct causality for reproductive outcomes; however, they underscore the necessity for a comprehensive evaluation of widespread sugar exposure, extending beyond traditional toxicity measures, particularly for at-risk populations and critical developmental periods. Collectively, the findings endorse the subsequent model: erythritol exposure precipitates ovarian oxidative stress, leading to granulosa cell damage and follicular structural anomalies, in conjunction with diminished AMH signaling. Chronic stress, especially in F2, correlates with inhibited autophagy and diminished PI3K–p85 survival signaling, hindering follicle formation and fostering ovarian dysfunction akin to accelerated reproductive aging characteristics.

### 4.1 Key limitations and future direction

A key limitation of the current work is that autophagy was assessed predominantly using steady-state measurements, including LC3 protein expression, IF analysis, and transcriptional profiling of autophagy-related genes such as Lc3 and Atg5. Although these methods consistently demonstrated autophagy inhibition, especially in the F2 generation, they fail to comprehensively represent the dynamic characteristics of autophagic flux. Autophagy is a meticulously regulated, multistage process encompassing autophagosome production, maturation, and lysosomal degradation, wherein fluctuations in LC3 levels alone are unable to differentiate between compromised autophagosome synthesis and faulty degradation.

Further studies should employ more sophisticated approaches to directly evaluate autophagic flow. Co-localization studies utilizing LC3 with lysosomal markers such as LAMP1 and cargo adaptor proteins like p62 would elucidate autophagosome–lysosome fusion and cargo turnover. Moreover, employing tandem fluorescent reporters like the mRFP-GFP-LC3 test facilitates the differentiation between autophagosomes and autolysosomes, thereby permitting a more accurate assessment of autophagic development. These methodologies will elucidate if erythritol-induced changes in ovarian tissue signify defective autophagosome production or compromised lysosomal degradation. Integrating these tools in future research will enhance the molecular comprehension of autophagy dysregulation in transgenerational ovarian dysfunction.

## 5 Conclusion

The current work represents comprehensive evidence that prenatal exposure to erythritol alters ovarian architecture and molecular signaling across generations. Although systemic growth and fertility seemed unchanged, notable ovarian changes were detected, such as compromised follicle maturation, histological degeneration, induction of oxidative stress, decreased AMH expression, inhibition of autophagy signaling, and downregulation of PI3K–p85 in F2 ovaries. These data suggest that erythritol disrupts the equilibrium between follicular survival and degeneration pathways. The transgenerational effects found in F2 progeny indicate possible epigenetic or developmental reprogramming mechanisms. Erythritol exposure may collectively expedite ovarian failure by inducing oxidative stress that suppresses autophagy and PI3K signaling. These findings elicit significant concerns about the long-term reproductive safety of elevated erythritol intake and require more mechanistic and clinical studies.

## Data Availability

The original contributions presented in the study are included in the article/**Supplementary Material**. Further inquiries can be directed to the corresponding author.

## Supplementary material

Supplementary Table 1 Primers for real-time RT-PCR.
