# Supplementary material for: Gestational exposure to the artificial sweetener erythritol reprograms ovarian function through AMH suppression and oxidative stress-mediated disruption of autophagy and PI3K signaling
Source: Front Endocrinol (Lausanne). 2026 May 14;17:1829421. doi: 10.3389/fendo.2026.1829421 (PMC13218215; doi:10.3389/fendo.2026.1829421)
Supplement: Supplementary Table 1 — Primers for real-time RT-PCR. [file Table1.docx]

**Table S1.** Primers for real-time RT-PCR

| **Gene Symbol** | **Sequences** |
| --- | --- |
| ***GAPDH*** | Forward: GCATCTTCTTGTGCAGTGCC  Reverse: GATGGTGATGGGTTTCCCGT |
| ***Esr2*** | Forward: GAGCTGACACCATGT  Reverse: CAGTCCACATAGCACT |
| ***Insl3*** | Forward: CTCTCACAGCTCTCA  Reverse: CACACTGAGCCTACAT |
| ***Igf-1*** | Forward: CGCTGAGCTACAAGTC  Reverse: GGAGCTCTCTACATC |
| ***Cyp19*** | Forward: GCAACAGGAGCTATAGATGAAC  Reverse: AGGCACGATGCTGGTGATG3 |
| ***Gdf-9*** | Forward: GATGTGACCTCCCTCCTTCA  Reverse: GCCTGGGTACTCGTGTCATT |
| ***Pi3k*** | Forward: GATGTCTGCGTTAGGGCTTACC  Reverse: TCAGCATCATGGAGAACAGGAT |
| ***Akt*** | Forward: CTCATTCCAGACCCACGAC  Reverse: ACAGCCCGAAGTCCGTTA |
| ***Mtor*** | Forward: TGCCTTCACAGATACCCAGTAC  Reverse: AGGTAGACCTTAAACTCGGAC |
| ***Lc3*** | Forward: TGTTAGGCTTGCTCTTTTGG  Reverse: GCAGAGGAAATGACCACAGAT |
| ***Atg5*** | Forward: CCTGAAGACGGAGAGAAGAAGAG  Reverse: CGGGAAGCAAGGGTGTCAT |
| ***Amh*** | Forward: AACTGAGTGCGTTCCAGGAG  Reverse: AGTTTTCTTTGCGCGTCGTA |
| ***Casp3*** | Forward: GGTATTGAGACAGACAGTGG  Reverse: CAT GGGATCTGTTTCTTTGC |
| ***Ccnd2*** | Forward: CCTCACGACTTCATTGAGCA  Reverse: GTAGCACACAGAGCGATGA |
| ***Dnmt3*** | Forward: ACGCCAAAGAAGTGTCTGCT  Reverse: CTTGGCTATTCTGCCGTGTT |
| ***ACTB*** | Forward: AGCCATGTACGTAGCCATCC  Reverse: ACCCTCATAGATGGGCACAG |
